# Preoperative HE4, CA125 and ROMA in the differential diagnosis of benign and malignant adnexal masses

**DOI:** 10.1186/s13048-016-0254-7

**Published:** 2016-07-19

**Authors:** Katarzyna M. Terlikowska, Bozena Dobrzycka, Anna M. Witkowska, Beata Mackowiak-Matejczyk, Tomasz Kamil Sledziewski, Maciej Kinalski, Slawomir J. Terlikowski

**Affiliations:** Department of Food Science and Technology, Medical University of Bialystok, 37 Szpitalna Street, 15-295 Bialystok, Poland; Department of Obstetrics, Gynaecology and Maternity Care, Medical University of Bialystok, 15 Warszawska Street, 15-062 Bialystok, Poland; Department of Gynaecologic Oncology, Maria Sklodowska-Curie Memorial Bialystok Oncology Center, 12 Ogrodowa Street, 15-027 Bialystok, Poland; Department of Gynaecology and Obstetrics, Jedrzej Sniadecki Memorial Hospital, 15 Warszawska Street, 15-062 Bialystok, Poland; Department of Obstetrics, Gynaecology and Maternity Care, Medical University of Bialystok, Szpitalna 37, 15-295 Bialystok, Poland

**Keywords:** HE4, CA125, ROMA, Ovarian cancer

## Abstract

**Background:**

The aim of this study was to evaluate HE4, CA125 and ROMA in the preoperative differentiation benign ovarian diseases from epithelial ovarian cancer depending on the menopausal status.

**Methods:**

In order to estimate markers’ concentrations in the serum of women with benign ovarian disease (*n* = 128) and with epithelial ovarian carcinoma (*n* = 96) the electrochemiluminescence (ECLIA) technique has been applied.

**Results:**

Using the ROC analysis, although no statistical differences were found among their AUCs, the ROMA algorithm seems to be effective in gathering the diverse performance of HE4 and CA125. The AUC for HE4, CA125 and ROMA for all patients were: 0.895; 0.879 and 0.918, respectively. At established new optimal cutoff values for HE4, CA125 and ROMA we found higher specificity in postmenopausal compared to premenopausal women (96.9 vs 89.8 % and 97.7 vs 84.1 % and 95.9 vs 89.1 %, respectively). The sensitivity of HE4 in pre- and postmenopausal women was similar (83.5 vs 83.8 %), while for CA125 was the highest in premenopausal women (87.0 vs 84.1 %). For HE4, CA125 and ROMA the negative predictive value was high (97.6, 93.9 and 94.4 %, respectively).

**Conclusions:**

The ROMA algorithm shows the best diagnostic performance to distinguish epithelial ovarian cancer from benign ovarian disease. We found the high specificity of HE4 and CA125 while differentiating ovarian benign diseases from epithelial ovarian cancer in postmenopausal women and the high sensitivity of CA125 in detecting epithelial ovarian cancer in premenopausal patients.

## Background

Ovarian cancer is the most lethal cancer among gynaecological malignancies. In 2012, it was estimated that 238,719 cases were diagnosed and 151,905 women died from this disease worldwide [1]. The estimated number of new ovarian cancer cases in Europe in 2012 was 65,538 with 42,704 deaths [2]. In Poland, ovarian cancer is the second most frequent invasive malignancy of the female genital tract after cancers of the uterine corpus, with an estimated 3,600 cases diagnosed annually. Approximately 2,600 women die each year from ovarian cancer, representing the most common cause of death among women with gynaecological malignancies [3].

Taking into consideration the late stage of diagnosis, our inability to diagnose ovarian cancer at an early stage remains the major problem. Despite the acceptance regarding the influence of reproductive hormones on ovarian cancer risk and considerable advances in the understanding of epithelial ovarian carcinogenesis on a molecular level, there is no complete understanding of the biologic processes underlying malignant transformation of ovarian surface epithelium [4–6]. The contemporary diagnostic standard of ovarian cancer includes transvaginal ultrasound and serum measurement of CA125. However this method has insufficient specificity, especially in women before menopause. A wide range of diagnostic approaches e.g. panels of biomarkers, algorithms, ultrasound and other imaging methods are being investigated at present [7, 8]. Among them the usefulness of HE4 in the diagnosis of ovarian cancer has been researched by a few groups [9–13].

Both CA125 and HE4 with menopausal status are currently being incorporated into the Risk of Ovarian Malignancy Algorithm (ROMA) in order to discern malignant from benign pelvic masses. Many studies have demonstrated the clinical utility of serum HE4 and ROMA in women with a complex pelvic mass and may provide even greater accuracy in the risk stratification of epithelial ovarian carcinoma [10, 11, 13, 14].

This study aimed to evaluate HE4, CA125 and ROMA in the preoperative differentiation benign ovarian diseases from epithelial ovarian cancer depending on the menopausal status.

## Methods

The analysis of the preoperative serum concentrations of CA125, HE4 and the ROMA values was performed on the material obtained from the Caucasian women surgically treated from 2012 to 2016 at the Department of Gynaecologic Oncology of the Maria Sklodowska-Curie Memorial Bialystok Oncology Centre and at the Department of Gynaecology and Obstetrics of the Jedrzej Sniadecki Memorial Hospital in Bialystok (Poland). It was done on account of benign ovarian disease (*n* = 128) and epithelial ovarian cancer (*n* = 96) according to the international treatment guidelines for ovarian cancer patients, including primary cytoreductive surgery followed by platinum-containing chemotherapy [15].

All surgical specimens were reviewed by 2 dedicated gynaecologic pathologists. Tumours were classified according to the WHO histological criteria [16] and divided into subtypes: serous 59 (61.5 %), mucinous 10 (10.4 %), endometrioid 13 (13.5 %), clear cell 5 (5.2 %), others (mixed non-differentiated) 8 (8.3 %) and no data 1 (1.1 %). The samples were grouped by the following histological grades: 5 (5.2 %) were classified as low-grade (G1), 10 (10.4 %) were medium-grade (G2) and 78 (81.3 %) were in high-grade (G3). Most of the epithelial ovarian cancers were of high grade and were diagnosed at an advanced stage. A total of 24 (25 %) were diagnosed with stage I disease, 11 (11.5 %) with stage II, 48 (50 %) with stage III and 12 (12.5 %) with stage IV disease, according to the Fédération Internationale de Gynécologie et d'Obstétrique (FIGO) classification criteria [17]. The benign disease group with serous, mucinous, endometriosis, mixed and other cysts of the ovary was also examined histologically. The women were made familiar with procedures and gave their written consent before the study enrolment. The study was approved by the Bioethics Committee of the Medical University of Bialystok (protocol No. R-I-002/68/2012).

Blood samples were collected to Vacutainer sterile tubes (Becton Dickinson, USA) to clot. Samples were then centrifuged for 10 min at 3000 rpm. Recovered sera were pipetted into sterile tubes (Nunc, Denmark) and stored at −80 °C. Concentrations of HE4 and CA125 were assessed with the electrochemiluminescence (ECLIA) technique on Cobas e411 (Roche Diagnostics, Switzerland) analyser, based on standard protocols. Cutoff levels were 35 U/mL for CA125 and 140 pmol/L for HE4. The range of HE4 and CA125 assays were 15–1500 pmol/l and 0.600–5000 U/ml, respectively. The test precision for both markers was performed in accordance with the protocol guidelines of Clinical and Laboratory Standards Institute (CLSI) [18]. All test runs were duplicated. According to the indications of the manufacturer, an index of ROMA ≥11.4 and ≥29.9 % indicates a high risk for the presence of epithelial ovarian cancer in pre- and postmenopausal women, respectively. The patients’ clinical status was not known by the people carrying out the assays, and the results of these assays were disclosed to the surgeons only after the patients’ disease status was recorded.

### Statistical analysis

ROMA classifies patients as being at a low or at a high risk for malignant disease using the following algorithms:

- Before menopause: PI = ‐ 12.0 + 2.38 × LN [HE4] + 0.0626 × LN [CA125]
- After menopause: PI = ‐ 8.09 + 1.04 × LN [HE4] + 0.732 × LN [CA125]
- ROMA value (%) = ePI/[1 + ePI] x 100%
- PI - predictive index, LN - natural logarithm, e - base of natural logarithm

The HE4, CA125 and ROMA median values were compared with the Mann-Whitney *U* test, and the Kruskal-Wallis one-way analysis of variance by ranks (ANOVA) and the Spearman’s rank correlation coefficient (rho). Categorical variables were compared with the Pearson’s chi-square test with Yates correction and Fisher’s exact test based on the Statistica software package 10.0 PL (StatSoft, Inc. StatSoft Poland Ltd.). Receiver operator characteristic (ROC) curves were constructed, and the area under the curve (ROC-AUC) with a 95 % confidence interval (95%CI) was calculated. Sensitivity and specificity were calculated in pre- and postmenopausal women separately and independently of menopausal status. In order to identify patients with cancer, the best cutoff point of CA125, HE4 and ROMA with regard to best values of sensitivity, specificity, positive (PPV) and negative predictive values (NPV) were evaluated. For all statistical comparisons, a *p*-value of <0.05 was considered statistically significant.

## Results

Of the 224 evaluable women, 120 were premenopausal (age: median: 36, range: 25–49) and 104 postmenopausal (age: median: 63, range 53–74 years). The clinical characteristics information of individuals enrolled in our study were shown in Table 1. High serum levels of HE4, CA125 and values of ROMA were found in patients with epithelial ovarian cancer rather than in those with benign diseases (*p* < 0.001). The total median value of HE4, CA125 and ROMA in pre- and postmenopausal women with epithelial ovarian cancer was statistically higher than that in the women with benign diseases (*p* < 0.001). HE4, CA125 and ROMA values determined in pre- and postmenopausal women with benign diseases and with epithelial ovarian cancer are shown in Table 2.

**Table 1:** Clinicopathological characteristics of the patients

Pathology	All patients *n* (%)	Menopausal status	
		Pre- n (%)	Post- n (%)
Benign ovarian diseases	128	87	41
* Histologic type*			
Serous	46 (35.9)	25 (28.7)	21 (51.2)
Mucinous	13 (10.2)	7 (8.1)	6 (14.6)
Endometriosis	33 (25.8)	31 (35.6)	2 (4.9)
Mixed	12 (9.4)	8 (9.2)	4 (9.8)
Other	24 (18.7)	16 (18.4)	8 (19.5)
Epithelial ovarian cancer	96	33	63
* Histologic type*			
Serous	59 (61.5)	17 (51.4)	42 (66.7)
Endometrioid	13 (13.5)	5 (15.2)	8 (12.7)
Mucinous	10 (10.4)	6 (18.2)	4 (6.3)
Clear cell	5 (5.2)	3 (9.1)	2 (3.2)
Mixed/Undifferentiated	8 (8.3)	2 (6.1)	6 (9.5)
No data	1 (1.1)	0 (0)	1 (1.6)
FIGO stage			
IA	5 (5.2)	3 (9.1)	2 (3.2)
IB	4 (4.1)	2 (6.1)	2 (3.2)
IC	15 (15.6)	8 (24.3)	7 (11.1)
IIA	2 (2.1)	0 (0)	2 (3.2)
IIB	1 (1.1)	1 (3.0)	0 (0)
IIC	8 (8.3)	3 (9.1)	5 (7.9)
IIIA	3 (3.1)	1 (3.0)	2 (3.2)
IIIB	9 (9.4)	1 (3.0)	8 (12.7)
IIIC	36 (37.5)	11 (33.3)	25 (39.6)
IV	12 (12.5)	2 (6.1)	10 (15.9)
not staged	1 (1.1)	1 (3.0)	0 (0)
Grade			
G1	5 (5.2)	3 (9.1)	2 (3.2)
G2	10 (10.4)	6 (18.2)	4 (6.3)
G3	78 (81.3)	23 (69.7)	55 (87.3)
unknown	3 (3.1)	1 (3.0)	2 (3.2)

**Table 2:** The serum levels of HE4 and CA125 and ROMA values in the examined groups

	Benign ovarian diseases	Epithelial ovarian cancer	*p*-value
	Median (range)	Median (range)	
all patients			
HE4 (pmol/l)	53.4 (22.1–328.6)	118.4 (37.4–1921.4)	<0.001
CA125 (U/ml)	19.4 (4.2–150.3)	116.3 (14.4–3798.4)	<0.001
ROMA (%)	12.4 (0.8–42.6)	50.4 (21.8–99.1)	<0.001
premenopausal			
HE4 (pmol/l)	50.4 (22.1–98.4)	84.1 (37.4–762.4)	<0.001
CA125 (U/ml)	23.3 (4.2–150.3)	75.4 (14.4–706.2)	<0.001
ROMA (%)	10.2 (0.8–35.8)	28.6 (21.8–85.6)	<0.001
postmenopausal			
HE4 (pmol/l)	56.4 (32.8–328.6)	147.8 (54.9–1921.4)	<0.001
CA125 (U/ml)	12.3 (9.4–113.3)	168.7 (15.9–3798.4)	<0.001
ROMA (%)	14.6 (1.2–42.6)	72.2 (30.2–99.1)	<0.001

**Fig. 1:**
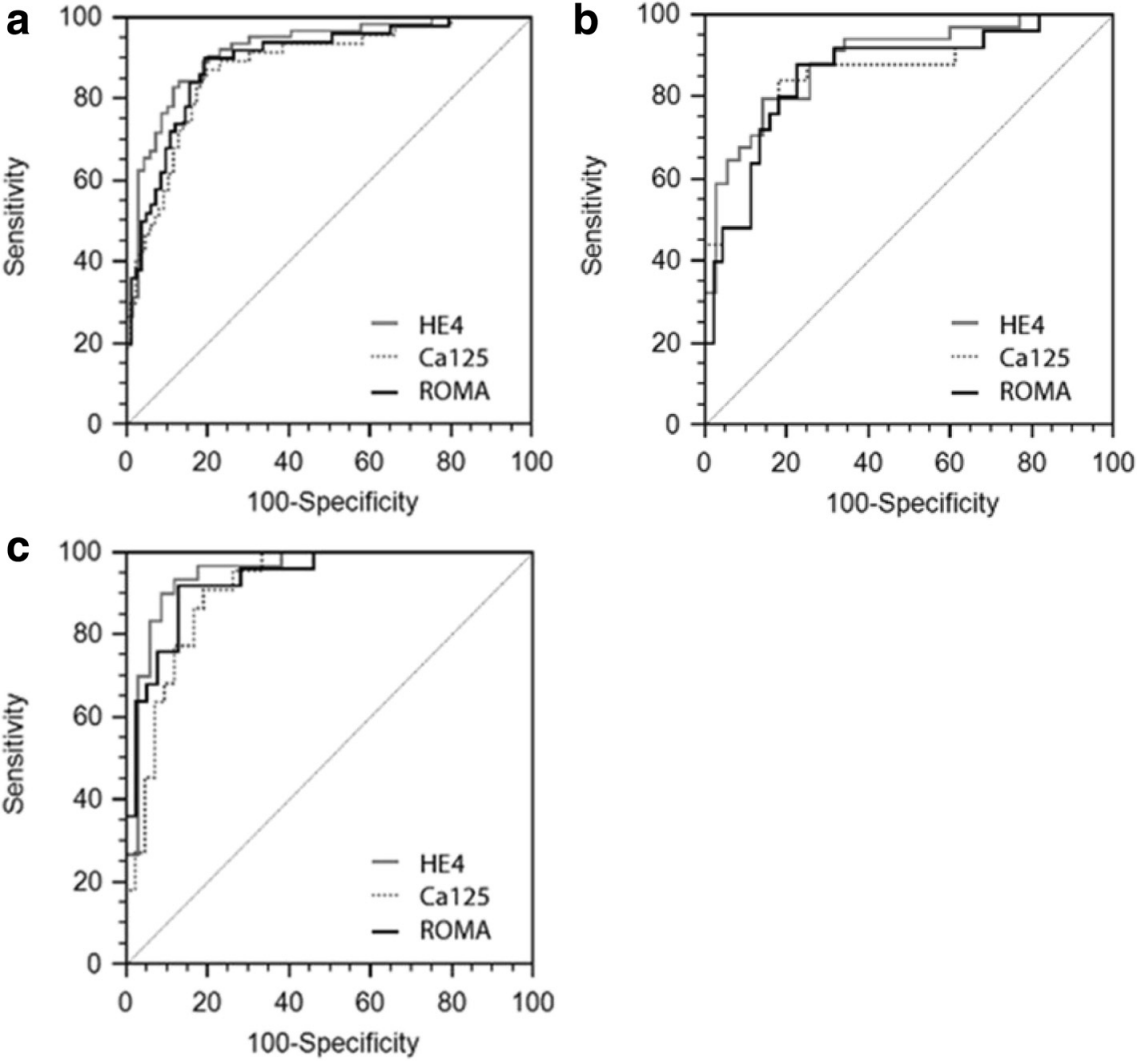
The ROC curves of HE4, CA125 and ROMA in all patients (**a**), premenopausal (**b**) and postmenopausal (**c**). Area under the ROC curve of HE4, CA125 and ROMA (**a**): 0.895 vs 0.879 vs 0.918, (**b**): 0.845 vs 0.833 vs 0.854 and (**c**): 0.916 vs 0.904 vs 0.931, respectively

In the present study we established new cutoff values specific to the examined population for each biomarker, and verified them using ROC analysis to calculate the optimal cutoffs. The best cutoff points distinguishing malignant vs. benign disease for HE4, CA125 and ROMA were 72.3 pmol/l; 62.2 U/ml and 20.1 %, respectively. Before and after the menopause these values were as follows: 70.3 vs 109.1 pmol/l for HE4; 64.6 vs 39.4 U/ml for CA125 and 14.9 vs 33.4 % for ROMA. The level of optimal cutoff values for HE4 was lower (72.3 pmol/l) than the recommended one (140 pmol/l), whereas for CA125 it was higher (62.2 U/ml) than the suggested one (35 U/ml), for all patients. The diagnosis accuracy of HE4, CA125 and ROMA was assessed by estimating ROC and AUC for all patients with ovarian cancer versus benign diseases. The AUC values for HE4, CA125 and ROMA were 0.895 (confidence interval (CI) 95 %, 0.838–0.951), 0.879 (CI 95 %, 0.818–0.941) and 0.918 (CI 95 %, 0.853–0.938), respectively. The highest ROC-AUC was for ROMA, followed by HE4. When alternative thresholds of 72.3 pmol/l (all), 70.3 pmol/l (premenopausal) and 109.1 pmol/l (postmenopausal) for HE4 were used, the sensitivities of HE4 in detecting epithelial ovarian cancer were enhanced, by 84.1 % (all), 83.5 % (pre-) and 83.8 % (post-), with just a slight loss of specificities from 97.5 to 86.3 % (all) and 98.6 to 89.8 % (pre-), respectively. In postmenopausal women specificity of HE4 rose from 94.2 to 96.9 %. The PPV for HE4 was 45.2 %, 46.2 and 86.8, respectively. The NPV was 97.6, 96.7 and 89.6 %, respectively. While in the case of CA125, the sensitivities obtained by using a modified cutoff value were significantly lower than when using the preferred one in postmenopausal group. The specificities were elevated to 82.4 % (all), 84.1 % (pre-) and 97.7 % (post-). The PPV for CA125 was 41.6, 44.8 and 91.7 % and the NPV was 93.9, 94.8 and 92.8 %, respectively. In the analysed groups no clear differences were found for ROMA values between optimal and preferred value settings (Table 3, Fig. 1).

**Table 3:** Diagnostic accuracy for discriminating between benign ovarian diseases and epithelial ovarian cancer

BOD vs EOC	Marker	ROC-AUC (95 % CI)	Cutoff optimal/preferred	Sensitivity (%) optimal/preferred	Specificity (%) optimal/preferred	PPV (%) optimal/preferred	NPV (%) optimal/preferred
all	HE4	0.895 (0.838–0.951)	72.3 / 140 (pmol/l)	84.1 / 67.1	86.3 / 97.5	45.2 / 87.4	97.6 / 92.1
	CA125	0.879 (0.818–0.941)	62.2 / 35 (U/ml)	83.1 / 81.9	82.4 / 74.1	41.6 / 36.9	93.9 / 96.2
	ROMA	0.918 (0.853–0.938)	20.1 / 11.4; 29.9 (%)	86.2 / 84.8	86.8 / 88.2	39.4 / 36.3	94.4 / 96.6
pre-menopausal	HE4	0.845 (0.806–0.894)	70.3 / 140 (pmol/l)	83.5 / 68.2	89.8 / 98.6	46.2 / 86.4	96.7 / 94.4
	CA125	0.833 (0.753–0.944)	64.6 / 35 (U/ml)	87.0 / 86.6	84.1 / 70.9	44.8 / 33.6	94.8 / 96.1
	ROMA	0.854 (0.778–0.876)	14.9 / 11.4 (%)	86.8 / 86.2	89.1 / 88.8	42.1 / 39.6	97.2 / 98.7
post-menopausal	HE4	0.916 (0.841–0.979)	109.1 / 140 (pmol/l)	83.8 / 87.1	96.9 / 94.2	86.8 / 92.8	89.6 / 86.2
	CA125	0.904 (0.855–0.943)	39.4 / 35 (U/ml)	84.1 / 91.9	96.7 / 89.8	91.7 / 83.2	92.8 / 94.4
	ROMA	0.931 (0.898–0.959)	33.4 / 29.9 (%)	89.0 / 86.8	95.9 / 92.2	91.9 / 89.4	94.2 / 94.9

*BOD* benign ovarian diseases, *EOC* epithelial ovarian cancer

## Discussion

The use of serum markers in a ovarian carcinoma risk classifier is novel but is strongly supported by literature [19]. Since HE4 is overexpressed in ovarian cancers relative to normal tissues, Hellstrom et al. [20] examined the potential of HE4 as a secreted biomarker for ovarian cancer. Studies performed by Bon et al. [21] showed the possibility of using HE4 in ovarian cancer diagnostics. The HE4 serum levels in healthy women have been reported to range from 60 pmol/l to 150 pmol/l. The reasons for this wide range might be due to the relationship between increasing HE4 serum levels and increasing age. Women older than 49 years of age have higher concentrations in comparison with women younger than 40 years. There is a correlation between the histological type and the serum concentration of HE4 with higher concentrations in serous ovarian cancer and with concentrations being lowest in patients with mucinous ovarian carcinomas [22–24].

Postmenopausal women with CA125 concentrations greater than 35 kU/L should be taken into consideration for transvaginal ultrasound examination as well as a computed tomography scan. The CA125 concentrations greater than 95 kU/L were reported to discriminate malignant from non-malignant pelvic masses with a positive predictive value of 95 % [25]. For premenopausal women, the American College of Obstetrics and Gynecologists suggested that patients with a pelvic mass and CA125 concentrations greater than 200 kU/L should be referred to a gynaecologist for consultation [19, 26].

In our recent study we showed that in the early stage of endometrioid endometrial cancer, HE4 can serve as a preoperative tool that can help to identify postmenopausal women who may require lymphadenectomy [27]. The present study aimed at evaluating and comparing the role of HE4, CA125 and ROMA value for the differential diagnosis of epithelial ovarian cancer. A cohort of women with benign ovarian diseases was used as the control group. We found that serum concentrations of HE4, CA125 and the ROMA values were significantly elevated in women with epithelial ovarian cancer compared to women with benign disease, both before and after the menopause, being similar to the results from other studies [28]. The data obtained from our study suggests that ROMA algorithm appears to have the best diagnostic performance in distinguishing epithelial ovarian cancer from benign ovarian diseases. Actually, using the ROC analysis while no statistical differences were found among their AUCs, the ROMA algorithm turns out to be effective in recognising the diverse performance of HE4 and CA125. The AUC for HE4, CA125 and ROMA for all patients were: 0.895; 0.879 and 0.918, respectively. Our results fit the range obtained by other authors: 0.85–0.96; 0.81–0.95 and 0.88–0.97 [20, 22, 29]. It is worth realising that the ROC analysis identifies optimal cutoffs which are different from the recommended routinely used ones for HE4 and CA125, insinuating the possibility of adjusting the cutoff points to the clinical requirements of a given diagnostic setting, as it was formerly reported [30–32]. Therefore, we decided not to use the limits specified by the assay procedures. The values were determined on the basis of the highest accuracy (minimal false negative and false positive results) and cutoffs were calculated depending on the menopausal status. Both in pre- and postmenopausal women, HE4, CA125 and ROMA values acquired similar sensitivity. However, in postmenopausal women all tested markers were characterized by higher specificity. For HE4, CA125 and ROMA the negative predictive value was high. When using the recommended cutoff points indicated by the manufacturers, HE4 yields the best specificity performances in both pre- and postmenopausal patients while CA125 displays the best sensitivity.

Van Gorp et al. [33], supplied insufficient sensitivity of HE4. The cutoff value of HE4 (150 pmol/l) in this study was based on the manufacturer’s protocol. HE4 cutoff point of 70 pmol/l used by Moore et al. [34], achieved 74.5 % of sensitivity and 83.3 % of specificity. Our study showed that the best cutoff point for HE4 was 72.3 pmol/l and depicted 84.1 % of sensitivity and 86.3 % of specificity. The best cutoff values for ROMA in pre- and postmenopausal women, set in the present study, were slightly higher from those suggested by other authors [35, 36]. Moore et al. [34] found higher sensitivity and specificity of ROMA in premenopausal compared to postmenopausal women (92.3 and 75.0 % vs 76.5 and 74.8 %, respectively). The cutoff values for ROMA were: 13.1 and 27.7 %, respectively. ROMA calculation enabled 93.8 % of ovarian cancer cases to be correctly classified as a high-risk group. For cutoff values suggested by Moore et al. [34] and Bandiera et al. [37] demonstrated 84.6 % of sensitivity and 81.2 % of specificity. Similar results were outlined in a multicentre prospective study from six countries in Asia [38]. In our study, at established new optimal cutoff values for ROMA, we found higher sensitivity and specificity in postmenopausal compared to premenopausal women (89.0 and 95.9 % vs 86.8 and 89.1 %, respectively). ROMA also showed significant difference in comparison with HE4 and CA125 for discriminating benign ovarian diseases and epithelial ovarian cancer in all the patients and postmenopausal women. The differences between values of sensitivity and specificity probably emerge from variations between the studied groups (different types of epithelial ovarian cancers and the number of investigated cases). Furthermore, our cutoff values were not set at 75 % level of specificity, but we estimated them based on the ROC curve points. Thus, these results need to be confirmed by more well-designed research studies.

## Conclusions

In conclusion, this study confirms the diagnostic role of HE4 and ROMA in epithelial ovarian cancer. The ROMA algorithm appears to show the best diagnostic performance to differentiate epithelial ovarian cancer from benign ovarian disease. We found the high specificity of HE4 and CA125 while discriminating ovarian benign diseases from epithelial ovarian cancer in postmenopausal women and the high sensitivity of CA125 in detecting epithelial ovarian cancer in premenopausal patients. Concisely, our study shows that ROMA algorithm more accurately selects patients with a high risk of ovarian epithelial cancer which enables to direct them to centres specializing in oncological gynaecology.

## Abbreviations

CA125: carbohydrate antigen 125; HE4: human epididymis protein 4; ROMA: Risk of Ovarian Malignancy Algorithm; FIGO: Fédération Internationale de Gynécologie et d'Obstétrique; ECLIA: electrochemiluminescence; CLSI: Clinical and Laboratory Standards Institute; PI: predictive index; LN: natural logarithm; e: base of natural logarithm; ROC: receiver operator characteristic; ROC-AUC: area under the curve; PPV: positive predictive values; NPV: negative predictive values; CI: confidence interval.
